# Clinical characteristics and the associated risk factors of the development of bilateral breast cancers: A case-control study

**DOI:** 10.1016/j.amsu.2020.10.064

**Published:** 2020-11-04

**Authors:** Sumadi Lukman Anwar, Dayat Prabowo, Widya Surya Avanti, Ery Kus Dwianingsih, Wirsma Arif Harahap, Teguh Aryandono

**Affiliations:** a Division of Surgical Oncology, Department of Surgery, Dr Sardjito Hospital/Faculty of Medicine, Public Health, and Nursing, Universitas Gadjah Mada, Yogyakarta, 55281, Indonesia; b Department of Radiology, Dr Sardjito Hospital/Faculty of Medicine, Public Health, and Nursing, Universitas Gadjah Mada, Yogyakarta, 55281, Indonesia; c Department of Anatomical Pathology, Dr Sardjito Hospital/Faculty of Medicine, Public Health, and Nursing, Universitas Gadjah Mada, Yogyakarta, 55281, Indonesia; d Division of Surgical Oncology, Department of Surgery, Dr M Jamil Hospital/Faculty of Medicine Universitas Andalas, Padang, 25127, Indonesia

**Keywords:** Bilateral, Contralateral, Breast cancer, Risks, Survival, Outcome

## Abstract

**Background:**

The clinical impacts of bilateralism on prognosis and clinical decision-making remain contradictory particularly in areas with low incidence and delayed diagnosis of primary breast cancer. Identification of women at risk of bilateral breast cancer is required to improve patient management and to design the appropriate surveillance.

**Methods:**

A total of 1083 women were enrolled and analyzed for the presence of synchronous and metachronous bilateral breast cancer as cases and unilateral breast cancer as controls during the median follow-up of 4.8 years.

**Results:**

The incidence of bilateral breast cancer was 7.5% (81 of 1083). In comparison with unilateral breast cancers, bilateral cases were significantly diagnosed in younger women (*P* = 0.037, mean age was 35.6 years) who had a larger tumor size (*P* = 0.012, mean tumor size was 8 cm in diameter). Histological type of lobular cancer was identified as one of the risk factors for the development of contralateral breast cancer (OR 5.564, 95% CI: 3.219–9.620) and synchronous bilateral breast cancer (OR 2.561, 95% CI: 1.182–5.550). Bilateral breast cancer had significantly shorter progression-free survival (Mean survival was 26.6 vs 52.5 months for bilateral and unilateral breast cancers, respectively; *P* = 0.001) and shorter time to develop distant metastasis (Mean survival was 41.7 vs 104 months for bilateral and unilateral breast cancers, respectively; *P* = 0.001).

**Conclusion:**

Patients with first primary breast tumors with lobular histological type and advanced stages were observed to have higher risks for the development of contralateral breast cancers.

## Highlights

•Lack of available data of risks of bilateral breast cancer in non-Caucasians.
• Identification of risks of contralateral breast cancer is useful to design surveillance.
• Lobular type is a significant risk for the development of contralateral breast cancer.

## 1 Introduction

Around 2–5% of breast cancer patients will develop contralateral cancer during their entire lifetime [1,2]. The risk of developing contralateral cancer is around 5-fold higher in patients with first primary breast cancer in comparison to healthy women [3]. Several studies have shown that the incidence of bilateral breast cancer in Western countries has increased [1,4] and is associated with the implementation of screening and early detection program [4,5]. However, screening implementation has been associated with a higher number of false positive findings particularly in premenopausal and nulliparity women [6] that might subsequently cause unnecessary anxiety. There is still a lack of information regarding bilateral breast cancer from countries without a breast cancer screening program including in Indonesia [7]. Women with lower social-economic status and living in rural areas have been associated with more advanced stages at diagnosis [8] and they might also have higher risks for the development of the contralateral breast cancer.

The impacts of bilateral breast cancer on adverse prognosis such as disease recurrence, distant metastasis, and overall survival have been reported with conflicting results [9,10]. Because appropriate treatment and surveillance are required to reduce the risk and potential adverse prognosis, understanding of the clinical course of bilateral breast cancer is very important [11]. In addition, the best practices including type of surgery and systemic treatment suitable for patients with a high-risk to develop bilateral breast cancer are not well-established.

Several studies have reported some factors associated with an increased risk of developing bilateral cancer such as a positive family history, a lobular histology type, and multicentric nodules [4,5,12]. Most studies assessing incidence and associated risk factors of bilateral breast cancer involve Caucasian patients with particular characteristics of older age (median age of more than 50 years) who are predominantly diagnosed at early stages [1,4,12]. Relatively few studies have addressed the incidence and clinical course of bilateral breast cancers in populations with younger ages and diagnosed at late stages. Identification of patients with a high risk to develop bilateral breast cancer is very important to determine the best preventive approach and surveillance plan for early detection of the contralateral cancer. In this study, demographic, reproductive, clinical, and pathological variables attributed to bilateral breast cancers were analyzed in comparison to unilateral cases among Indonesian women. Treatment patterns and the association of bilateral breast cancer with risk of distant metastasis and disease progression were also studied. This study was conducted and reported according to the STROCSS guidelines [13].

## 2 Materials and methods

### 2.1 Cohort of breast cancer patients

A cohort of breast cancer patients diagnosed and treated at the Department of Surgery, Dr. Sardjito Hospital in 2013–2018 was queried for all primary breast cancers with unilateral cancers as a control and bilateral breast cancer as a case. Metastatic breast cancers at diagnosis were excluded. Bilateral breast cancers were differentiated with metastatic manifestations according to Chaudary's definition [14] including presentation of *in situ* tumor, different tumor grades and histological types, and the absence of locoregional or distant metastasis. Disseminated lesions beyond the axillary lymph nodes are considered as a high-risk of metastatic manifestation in the contralateral breast. Therefore, patients with metastatic lesions and disseminated lesions beyond the axillary lymph nodes were not included in this study [5]. The bilateral breast cancers were classified as synchronous if the contralateral cancer was definitely diagnosed within 12 months and metachronous if the contralateral cancer was definitely diagnosed after 12 months from the initial primary cancer [5]. The first breast cancer was determined as the initial tumor firstly diagnosed and the second cancer was the contralateral tumor which was diagnosed after the first breast cancer. All patients were clinically managed following the national guidelines from the Indonesian Society of Surgical Oncology and the local hospital protocols. Follow-up was performed every month for the first 6 months, every three months for the first year, and every 6 months for 5 years. Follow-up was performed annually after the first 5 years. This study was reviewed and approved by the Ethics Committee of the Medical Faculty – Universitas Gadjah Mada, Indonesia (1143/EC/2017 and 1049/EC/2018).

### 2.2 Data mining

Demographic and clinicopathologic data including the patient's age, tumor size, stage, histological type, axillary lymph node involvement, and presence or absence of distant metastasis at the initial primary breast cancer (first tumor) and the contralateral cancer (second cancer) as well as administered treatment were collected from the medical records. Grouping of stages, histological type, tumor grade, immunohistochemistry of hormonal receptors, breast cancer subtypes as well as demographic variables was performed as previously reported [8].

Progression-free survival was calculated from the time of initial diagnosis of breast cancer to the presence of any locoregional relapse or distant metastasis or cancer-related mortality. Time to distant metastasis was calculated from the initial diagnosis of breast cancer to the presence of cancer dissemination to the bone, lung, liver and brain as indicated by clinical manifestations and/or confirmed with imaging/pathology examination.

### 2.3 Statistical analysis

The associated risk factors for developing bilateral breast cancer were compared between unilateral and bilateral breast cancers as well as between synchronous and metachronous cancers. Categorical variables were compared with χ2 or Fisher-exact tests and continuous variables were analyzed using Mann-Whitney-U tests. The association with bilateral breast cancer status was analyzed using univariable and multivariable logistic regression. Survival analysis of recurrence-free and time to metastasis was performed using Kaplan-Meier curve and Mantel-Cox tests. The SPSS 17.0 software (SPSS Inc., Chicago) was used for all statistical analysis. All statistical analyses were performed with two-sided tests and *P*-value less than 0.05 indicated a statistically significant difference with 95% confidence interval (CI).

## 3 Results

### 3.1 Histological, staging, and hormonal status differences in bilateral breast cancer

Of the 1083 non-metastatic breast cancers, 81 patients (7.5%) developed bilateral breast cancer during median follow-up of 4.8 years. Among patients with bilateral breast cancer, 46 cases (56.8%) were synchronous cancers and 35 cases (43.2%) were metachronous cancers. The mean and median intervals for the development of metachronous bilateral breast cancers were 25 and 23 months, respectively. The diameter of secondary bilateral cancers was significantly smaller than their associated first tumors (means were 78.6 mm vs 55.8 mm, *P* = 0.001). In synchronous bilateral cancers, the first tumors were also significantly larger than the second tumors (82.28 mm versus 55.49 mm, *P* = 0.026).

The most common histology type in bilateral breast cancer was ductal carcinoma (63%, N = 51, Table 1). Compared with unilateral breast cancer, lobular type was significantly more frequent in bilateral breast cancer as well as in synchronous cancers (*P* = 0.001, respectively). The concordance of histological type and tumor differentiation between first and secondary cancer was 89.1% and 78.2, respectively in synchronous cancers; 88.5% and 77.1%, respectively in metachronous cancers. The secondary cancers were significantly more likely to be diagnosed with negative axillary lymph nodes than the first cancer in bilateral breast cancer (*P* = 0.023, Table 2). However, the first and secondary cancers were not significantly different in the clinical stages at diagnosis (*P* = 0.402, Table 2).

**Table 1:** Comparison of clinicopathological characteristics between unilateral and bilateral breast cancers.

Variables	UBC (N = 1002)	BBC (N = 81)		*P* value^a^	*P* value^b^	*P* value^c^
		First BC	Second BC			
Age (years)						
Mean	51.1	48.8	50.02	0.037*	0.222*	**0.0001**^#^
Median	51.0	47.0	47.0			
Tumor size (mm)						
Mean	62.73	78.6	55.8	0.012*	0.005*	**0.001**^#^
Median	60.0	62.0	50.0			
Histology						
Ductal	853	51 (63.0%)	56	**0.001**	**0.007**	0.722
Lobular	66	24 (29.6%)	20			
Other	83	6 (7.4%)	5			
Node status						
N0	283 (%)	18 (%)	45 (55.6%)	0.491	0.392	0.001
N1	504 (%)	44 (%)	17 (21.0%)			
N2	178 (%)	14 (%)	10 (12.3%)			
N3	37 (%)	5 (%)	9 (11.1%)			
Stage						
I	12 (%)	0 (0%)	16 (19.8%)	0.052	**0.001**	0.257
II	339 (%)	18 (22.2%)	32 (39.5%)			
III	651 (%)	63 (77.8%)	33 (40.7%)			
ER status						
Positive	561 (56%)	47 (58.0%)	45 (55.6%)	0.940	0.771	0.001
Negative	441 (44%)	36 (42.0%)	36 (44.4%)			
PR status						
Positive	434 (43.4%)	31 (38.3%)	24 (29.6%)	0.266	**0.017**	0.001
Negative	566 (56.6%)	50 (61.7%)	57 (70.4%)			
Her2 status						
Positive	275 (27.5%)	18 (22.2%)	18 (22.2%)	0.302	0.427	0.002
Negative	725 (72.5%)	63 (77.8%)	63 (77.8%)			
Surgery						
Mastectomy	901 (%)	77 (95.1%)	68 (84%)	0.006	0.412	0.001
BCT	97 (%)	2 (2.5%)	13 (16%)			
Biopsy	4 (%)	2 (2.5%)	0 (0%)			
Hormonal therapy						
Yes	572 (%)	47 (58.0%)	43 (53.1%)	0.317	0.853	0.001
No	430 (%)	34 (42.0%)	38 (46.9%)			
Chemotherapy						
Yes	915 (%)	76 (93.8%)	67 (82.7%)	0.438	0.028	0.804
No	87 (%)	5 (6.2%)	14 (17.3%)			
Radiotherapy						
Yes	744 (%)	61 (75.3%)	51 (63.0%)	0.834	0.032	0.017
No	258 (%)	20 (24.7%)	30 (37.0%)			

UBC: unilateral breast cancer.

BBC: bilateral breast cancer.

SBC: synchronous bilateral breast cancer.

MBC: metachronous bilateral breast cancer.

* Mann-Whitney u test.

# *t*-test.

a First cancer of unilateral vs. unilateral tumors.

b Second cancer of unilateral vs. bilateral tumors.

c First vs. second tumors of bilateral cancer.

**Table 2:** Comparison of clinicopathological characteristics between synchronous and metachronous bilateral breast cancers.

Variables	Synchronous bilateral (SBC) (N = 46)		Metachronous bilateral (MBC) (N = 35)		*P* value^a^	*P* value^b^	*P* value^c^	*P* value^d^
	First BC	Second BC	First BC	Second BC				
Age (years)								
Mean	49.2	49.48	48.5	50.7	0.001^#^	0.001^#^	0.985*	0.301*
Median	48.0	48.0	48.0	51				
Tumor size (mm)								
Mean	82.28	55.49	65.0	55.4	0.026^#^	0.062^#^	0.593*	0.927*
Median	70.0	50.0	60.0	50.0				
Histologi								
Ductal	25	29	27	27	0.001	0.001	0.021	0.146
Lobular	18	15	4	5				
Other	3	2	4	3				
Node status								
N0	8	29	10	16	0.566	0.013	0.054	0.278
N1	30	9	14	8				
N2	6	3	8	7				
N3	2	5	3	4				
Stage								
I	0 (0%)	8	0 (0%)	8	0.706	0.207	0.231	0.082
II	8 (%)	23	10 (%)	9				
III	38 (%)	15	25 (%)	18				
ER status								
Positive	28 (%)	22	20 (%)	23 (%)	0.295	0.624	0.552	0.109
Negative	18 (%)	24	15 (%)	12 (%)				
PR status								
Positive	18 (%)	12	14 (%)	12 (%)	0.266	0.805	0.855	0.423
Negative	28 (%)	34	21 (%)	23 (%)				
Her2 status								
Positive	10 (%)	11	8 (%)	7 (%)	1.00	1.00	0.905	0.675
Negative	36 (%)	35	27 (%)	28 (%)				
Surgery								
Mastectomy	43 (%)	38	34 (%)	30 (%)	0.422	0.679	0.452	0.706
BCT	2 (%)	8	0 (%)	5 (%)				
Biopsy	1 (%)	0	1 8%)	0 (0%)				
Hormonal therapy								
Yes	27 (%)	22	20 (%)	21 (%)	0.402	1.00	0.888	0.277
No	19 (%)	24	15 (%)	14 (%)				
Chemotherapy								
Yes	42 (%)	36	34 (%)	30 (%)	0.145	0.198	0.279	0.544
No	4 (%)	10	1 (%)	5 (%)				
Radiotherapy								
Yes	36 (%)	28	25 (%)	23 (%)	0.112	0.797	0.480	0.655
No	10 (%)	18	10 (%)	12 (%)				

*Mann-Whitney u test.

# *t*-test.

a Comparison of the first and second tumors in synchronous bilateral breast cancer.

b Comparison of the first and second tumors in metachronous bilateral breast cancer.

c Comparison of the first tumors between synchronous and metachronous bilateral breast cancer.

d Comparison of the second tumors between synchronous and metachronous bilateral breast cancer.

The expressions of estrogen receptors (ER), progesterone receptors (PR), and Her2 were not significantly different between unilateral and bilateral breast cancers. ER, PR, and Her2 expressions were significantly different between first and the contralateral tumors (*P* = 0.001, respectively). There was conversion of positive into negative expression and *vice versa* of ER and PR from first to the second cancer (Table 2). Concordance rates of first and second breast cancer were 84.7% and 71.4% for ER, 80.4% and 68.5% for PR, and 86.9% and 62.9% for HER2 in synchronous and metachronous bilateral breast cancers, respectively.

### 3.2 Risk factors of bilateral breast cancer

Advanced stages and tumor infiltration to the skin and chest wall were associated with risk of developing bilateral breast cancer (OR 1.887, 95% CI: 1.100–3.237), *P* = 0.021 and odds ratio (OR 1.746, 95% CI: 1.064–2.867, *P* = 0.028, respectively). The association was also observed in synchronous bilateral breast cancer (OR 9.980, 95% CI: 5.274–18.885, *P* = 0.001 and OR 2.086, 95% CI: 1.116–3.901, *P* = 0.021 for advanced stage and T4, respectively), (Table 3). However, only histological type of lobular carcinoma was associated with risks of developing bilateral breast cancer using both univariate (OR 5.971, 95% CI: 3.486–10.229, *P* = 0.001) and multivariate logistic regression analyses (OR 5.564, 95% CI: 3.219–9.620, *P* = 0.001). The association of lobular type with risk of developing synchronous bilateral breast cancer was also observed in both univariate and multivariate regression analyses (OR 9.980, 95% CI: 5.274–18.885, *P* = 0.001 and OR 8.878, 95% CI: 4.683–16.996, *P* = 0.001, respectively).

**Table 3:** Odds ratios and 95% confidence intervals of bilateral breast cancer, synchronous, and metachronous bilateral breast cancer in comparison to unilateral breast cancer using univariable and multivariable logistic regression.

Variables	Category	Bilateral breast cancer				Synchronous bilateral breast cancer											Metachronous bilateral breast cancer										
		OR	*P* value	OR (95% CI)	*P*-value		OR (95% CI)			*P* value		OR (95%CI)			*P* value			OR (95%CI)			*P* value		OR (95% CI)			*P* value	
		Univariate		Multivariate					Univariate					Multivariate						Univariate					Multivariate		
Age (years)	≤40	1.300 (0.743–2.272)	0.358	0.748 (0.412–1.358)				1.138 (0.501–2.588)			0.757		2.593 (0.952–7.063)			0.062			1.957 (0.923–4.149)			0.080		1.965 (0.535–7.194)			0.309
	>40	ref	ref		ref			ref					ref			ref			ref			ref		ref			
Menarche	≤12	1.171 (0.662–2.073)	0.578	1.163 (0.640–2.112)	0.620			1.002 (0.459–2.185)			0.996		1.082 (0.487–2.404)			0.846			1.410 (0.630–3.156)			0.403		1.481 (0.650–3.372)			0.350
	>12	ref	ref	ref	ref			ref					ref			ref			ref			ref		ref			
Menopause	≤50	ref		ref	ref			ref					ref						ref					ref			
	>50	0.593 (0.273–1.278)	0.186	0.572 (0.386–1.174)	0.163			0.677 (0.254–1.806)			0.436		0.512 (0.217–1.712)			0.448			0.492 (0.144–1.681)			0.257		0.511 (0.102–2.566)			0.511
Parity	Nulliparity	0.889 (0.417–1.894)	0.386	0.920 (0.322–2.632)	0.876			1.216 (0.505–2.933)			0.663		2.702 (0.602–12.048)			0.195			0.491 (0.116–2.075)			0.334		3.139 (0.607–16.219)			0.172
	Multiparity	ref	ref		ref			ref					ref			ref						ref		ref			
Breastfeeding	Yes	1.039 (0.588–1.833)	0.896	0.903 (0.410–1.990)	0.800			1.214 (0.558–2.643)			0.625		2.294 (0.613–8.587)			0.218			0.863 (0.386–1.927)			0.719		0.597 (0.237–1.507)			0.275
	No	ref	ref	ref	ref			ref			ref								ref					0.433–2.772			0.848
BMI	≥25	1.135 (0.716–1.800)	0.591	1.195 (0.724–1.974)	0.486			1.061 (0.579–1.945)			0.843		1.171 (0.627–2.189)			0.621			1.238 (0.626–2.448)			0.539		1.264 (0.625–2.559)			0.515
	<25	ref	ref	ref	ref			ref					ref			ref			ref					ref			
Family history	Yes	1.398 (0.816–2.398)	0.221	0.673 (0.386–1.174)	0.163			1.111 (0.527–2.342)			0.782		0.836 (0.384–1.819)			0.836			1.828 (0.862–8.621)			0.116		1.848 (0.871–3.922)			0.110
	No	ref	ref		ref			ref					ref			ref			ref					ref			
Grade	I-II	ref	ref	ref	ref			ref					ref			ref						ref		ref			
	III	0.933 (0.535–1.628)	0.807	0.912 (0.506–1.643)	0.758			0.789 (0.394–1.580)			0.503		0.64580.315–1.324)			0.233			1.198 (0.491–2.924)			0.692		1.072 (0.403–2.849)			0.890
Histology	Lobular	5.971 (3.486–10.229)	**0.001**	5.564 (3.219–9.620)	**0.001**			9.980 (5.274–18.885)			**0.001**		8.878 (4.683–16.996)			**0.001**			2.364 (0.888–6.292)			0.085		2.319 (0.863–6.233)			0.095
	Ductal and other	ref						ref																			
Stage	I-II	ref	ref		ref			ref					ref			ref						ref		ref			
	III	1.887 (1.100–3.237)	**0.021**	1.316 (0.657–2.638)	0.439			2.561 (1.182–5.550)			**0.001**		1.577 (0.595–4.117)			0.360			1.348 (0.640–2.839)			0.432		1.475 (0.458–4.749)			0.514
Tumor size	≤5	ref	ref	ref	ref			ref					ref			ref						ref		ref			
	>5	1.115 (0.683–1.819)	0.663	1.101 (0.533–2.274)	0.795			1.584 (0.794–3.158)			0.192		1.148 (0.538–2.449)			0.722			0.747 (0.375–1.487)			0.406		0.584 (0.271–1.262)			0.172
T	T1-2	ref	ref		ref			ref					ref			ref						ref		ref			
	T3-4	1.322 (0.751–2.327)	0.333	1.268 (0.623–2.580)	0.513			1.546 (0.711–3.358)			0.271		0.753 (0.288–1.965)			0.562			1.098 (0.492–2.449)			0.819		1.146 (0.483–2.717)			0.757
	T1-3	ref	ref		ref			ref					ref			ref						ref		ref			
	T4	1.746 (1.064–2.867)	**0.028**	1.308 (0.756–2.264)	0.337			2.086 (1.116–3.901)			**0.021**		1.377 (0.685–2.768)			0.369			1.354 (0.625–2.935)			0.442		1.200 (0.521–2.762)			0.669
N	N0	ref	ref	ref				ref					ref			ref						ref		ref			
	N1-3	1.655 (0.942–2.908)	0.080	1.277 (0.641–2.544)	0.487			1.879 (0.866–4.077)			0.111		1.294 (0.509–3.285)			0.588			0.989 (0.469–2.085)			0.977		1.274 (0.472–3.437)			0.633
Chemotherapy	Yes	1.445 (0.570–3.667)	0.438	1.384 (0.531–3.604)	0.506			0.998 (0.350–2.850)			0.998		1.384 (0.531–3.604)			0.506						ref		ref			
	No	ref	ref	**0.001**	0.417			ref					ref						3.233 (0.437–23.904)			0.437		1.143 (0.416–3.138)			0.796
Radiotherapy	Yes	1.058 (0.626–1.787)	0.834	0.683 (0.346–1.347)	0.271			ref					0.638 (0.346–1.347)			0.271						ref		ref			
	No	ref	ref	ref	1.923			1.248 (0.611–2.551)			0.543		ref						0.867 (0.411–1.830)			0.766		0.786 (0.305–2.027)			0.618
Surgery	MRM	2.158 (0.774–6.020)	0.142	1.480 (0.483–4.522)	0.491			1.607 (0.490–5.273)			0.434		1.480 (0.484–4.522)			0.491			3.811 (0.516–28.139)			0.190		3.866 (0.475–31.498)			0.206
	BCT and biopsy	ref	ref	ref				ref					ref			ref								ref			

### 3.3 Clinical management of bilateral breast cancers

Sixty-three (77.8%) patients with bilateral breast cancer were diagnosed in stage III, and 95% (N = 77) underwent mastectomy. In the contralateral cancers, 82% (N = 38) and 77% (N = 27) were diagnosed in the Stage II-III in synchronous and metachronous bilateral breast cancers, respectively. In addition, mastectomy was performed in 82% (N = 38) and 85.7% (N = 30) of the contralateral cancer in synchronous and metachronous cancers, respectively. Although mastectomy was more frequent in unilateral cancer, the difference was not significant compared with bilateral cancer (*P* = 0.142, Table 3). Chemotherapy was administered in 93.8% of patients with bilateral breast cancer when they were diagnosed with the first tumor and in 82.7% of them after diagnosis of the contralateral cancers. No significant differences were observed in the hormonal therapy and radiotherapy between unilateral and bilateral breast cancer (*P* = 0.317 and *P* = 0.834, respectively). All treatment modalities were delivered without any significant difference between unilateral and bilateral breast cancers (Table 1, Table 2). However, radiotherapy and hormonal therapy were more frequently delivered in the first cancer than after diagnosis of secondary cancer in patients with bilateral breast cancer (*P* = 0.017 and *P* = 0.001, respectively) as shown in Table 1.

### 3.4 Disease-free recurrence and time to metastasis

**Fig. 1:**
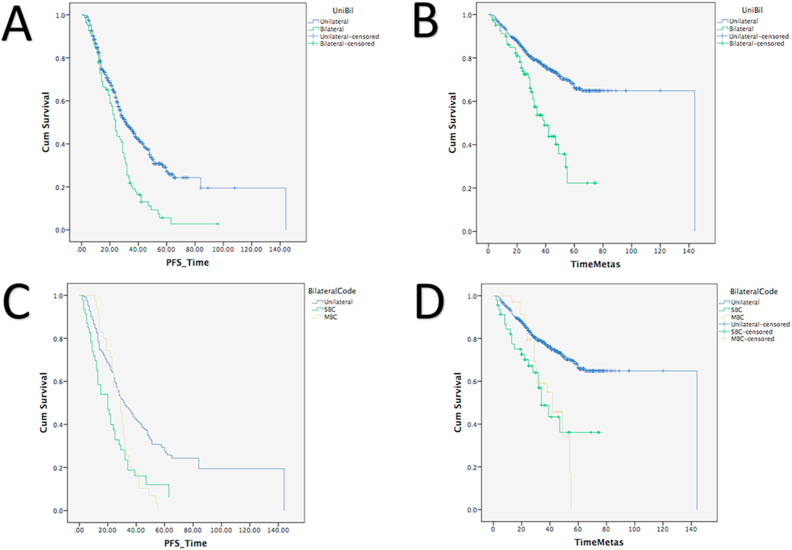
Association of bilateral breast cancer with worse prognosis compared to unilateral breast cancer. (A) Bilateral breast cancer had significantly shorter progression survival (means were 26.6 months in bilateral cancers and 52.5 months in unilateral cancers, Log-rank Mantel-Cox test, *P* = 0.001). (B) Bilateral breast cancer had significantly shorter time to develop distant metastasis (means were 41.7 months in bilateral cancer and 104.1 months in unilateral breast cancer, Log-rank Mantel-Cox test, *P* = 0.001). Synchronous and metachronous bilateral breast cancers had significant shorter progression free survival (Means were 25.6 and 28.7 months for synchronous and metachronous bilateral breast cancer, respectively; Log-rank Mantel-Cox test, *P* = 0.005 and *P* = 0.001, respectively; Panel C) as well as shorter time to distant metastasis (D) compared to unilateral breast cancer (Means were 42.4 and 40.0 months for synchronous and metachronous bilateral breast cancer, respectively; Log-rank Mantel-Cox test, *P* = 0.001, respectively). No significant differences in PFS and time to distant metastasis were found between synchronous and metachronous breast cancers.

In our study cohort, having bilateral breast cancer was associated with an increased risk of distant metastasis (OR 3.222, 95% CI: 2.034–5.098, *P* = 0.001). In comparison to unilateral breast cancer, bilateral breast cancer had significantly shorter progression-free survival (means were 26.6 vs 52.5 months, respectively; Log-rank Mantel-Cox test, *P* = 0.001), Fig. 1. Using Kaplan-Meier curve, bilateral cancer also showed significantly shorter time to progress into distant metastasis (means were 41.7 vs 104.1 months, respectively; Log-rank Mantel-Cox test, *P* = 0.001) (Fig. 1). Both synchronous and metachronous bilateral breast cancers had significantly poorer progression-free survivals (*P* = 0.001 and *P* = 0.005, respectively) as well as significantly shorter time to develop distant metastasis compared to unilateral breast cancer (*P* = 0.001 and *P* = 0.001, respectively) (Fig. 1). No significant differences were observed in the progression-free survivals and progression to distant metastasis between synchronous and metachronous breast cancers.

## 4 Discussion

In this study involving 1083 Indonesian patients, we identified incidence, risk factors, and prognosis of 81 (7.5%) patients with bilateral breast cancer. In the management of breast cancer, estimation of the risk for the development of contralateral breast cancer is very important to design an appropriate surveillance program [11]. Locoregional and systemic treatment with timely follow-up are performed to prevent locoregional recurrence, progression into a distant metastasis, as well as the development of contralateral breast cancer [11]. According to the clinical characteristics of primary cancer, patients with bilateral breast cancer were more frequently diagnosed in more advanced diseases, although the significant association was found only in the tumor size (T status, Table 3). Our last results might indicate the involvement of metastatic process of cancer cells in the development of contralateral cancer because the risk factors are overlapping with risks of distant spread as shown in our previous report [8,15]. However, some studies reported a lack of association between advanced tumor and risk of bilateral cancer [5,12,16]. Kheirelseid et al. reported smaller tumor size and earlier stage at diagnosis in bilateral compared to unilateral breast cancer [5]. Another study showed that high T- and N-status were more likely to be diagnosed with bilateral breast cancer within six months [17].

Our results showed high concordance (63–93%) of the histology, grades, and expression of hormonal receptor and HER2 between first and secondary cancers in the bilateral cases. Although histological grading and tumor histology are usually used to differentiate with a metastatic lesion, a second primary cancer often shares the similar histological characteristics with the first tumor [18]. ER positivity was associated with the risk for bilateral breast cancer [19], while another study showed the association of hormonal receptor negativity with bilateral breast cancer particularly in women with age younger than 50 years at diagnosis [20]. One study reported no association of bilateral cancer with hormonal receptor expression [5]. SEER data showed discordant ER expression in 10% of synchronous bilateral breast cancer and in 15% of metachronous bilateral breast cancer [21]. Our study showed no association of ER, PR, and HER2 expressions with bilateral breast cancer.

Several studies have revealed that younger age is a higher risk of the development of bilateral breast cancer [5,22,23]. In our study, patients with bilateral breast cancer were also initially diagnosed younger than those with unilateral breast cancer (Table 1). Using categorical variables, we identified that lobular type, advanced stage, and tumor infiltration to the skin and chest wall were associated with higher risk of bilateral cancer (Table 3). However, only histology of invasive lobular carcinoma was significantly associated with higher risk of bilateral breast cancer using multivariate analysis (Table 3). The association was also observed in synchronous but not in metachronous bilateral breast cancer (Table 3). Numerous studies have reported a higher risk of lobular histology for the development of contralateral breast cancer [17,24,25]. Lack of *CDH1* functions due to mutations, loss of heterozygosity, and promoter methylation has been associated with the underlying molecular mechanism of invasive lobular breast cancer [26]. E-cadherin preserves tissue structure and integrity through its natural properties as a transmembrane adhesion molecule [26,27]. Therefore, *CDH1* inactivation is related with multifocal cancer and the propensity of bilateral cancer [27]. Risk of bilateral breast cancer is reported higher in patients with a genetic predisposition [28]. Because the frequency of *BRCA1* and *BRCA2* mutations in Indonesia are relatively low [29] and the genetic tests were not widely available, our study did not specifically address the genetic risk for the development of bilateral cancer. Patients with germline *BRCA1* and *BRCA2* mutations have annual risk of 2–6% for developing contralateral breast cancer [28].

In our study, the second breast cancers were diagnosed with smaller tumor size (T) and nodal status (N) than the first cancers (Table 2). However, 40.7% of the secondary breast cancers were diagnosed in late stages and 44% were diagnosed with positive axillary lymph nodes. More studies are required to design surveillance strategies to improve early detection of contralateral cancer among patients with breast cancer in Indonesia. Mammography has been associated with lower sensitivity to detect contralateral cancer due to mammographically occult lesions in the contralateral breast, misinterpretation, perception error, and manifestations of lobular cancer as a focal architectural deformation or subtle asymmetric densities [30]. Intense surveillance program using additional magnetic resonance imaging (MRI) or breast ultrasonography for high risk women has resulted in higher detection rates accompanied with higher false-positive rates [31]. As the risk of bilateral breast cancer is relatively higher in younger patients, mode of detection other than breast ultrasonography and mammography such as MRI might be required for high risk patients younger than 35 years or with known *BRCA1/*2 mutations. Further studies are required to evaluate the cost-effectivity for incorporation of MRI in the surveillance program.

Mastectomy was the main type of surgery for both the first and second cancers because of late recognition and advanced stages at diagnosis (Table 2). Although general consensus on the optimal surgery approach for patients with bilateral breast cancer is not well established, recent studies have shown that bilateral mastectomy has been performed more frequently in synchronous bilateral cancer [5,32,33]. Clinical treatment of bilateral cancer including breast surgery has been suggested to be in line with unilateral breast cancer because the indication, contraindication, and prognosis are similar in patients with bilateral breast cancer treated with breast conservation surgery [32]. Our study showed that mode of initial surgery, radiotherapy, and systemic treatment were not significantly different between unilateral and bilateral breast cancers.

Bilateral breast cancer has been associated with poor prognosis compared to unilateral breast cancer [34,35]. Our study showed that patients with bilateral breast cancer had shorter time to relapse and to develop distant metastasis (Fig. 1). Younger age at diagnosis in bilateral cancers might also influence the prognosis as younger breast cancer patients often showed more aggressive clinical behavior and treatment resistance [23]. Younger patients might also have predisposing genetics for the development of contralateral cancer [1,4]. Although systemic therapy has been associated with reduced risk of contralateral cancer [3,36], the treatment is also suggested to have adverse effects on the biology of the secondary cancer [35]. However, there is also the probability that poor prognosis of bilateral cancer might be due to misclassification of metastatic lesions in the contralateral breast due to difficulty in the differentiation with second primary cancer. In our study, we excluded diagnosis of bilateral cancer in the presence of distant metastasis and locoregional relapses. In addition, the increasing risk for the development of contralateral cancer and treatment for bilateral breast cancer also often cause emotional and physical distress [37]. Concerns of contralateral cancer development, fear of disease recurrence, anxiety, body image mood disorders are commonly experienced by all patients with breast cancer [[37], [38], [39]]. In addition to new approaches for surveillance in high risk patients, enhancing coping strategies to adapt to a life with uncertainty might also be needed [37,39]. For women with difficulty in coping and adaptation, psychological support and intervention might also be required [37,40].

The main strength of this study is that it is the first study reporting the associated risks of bilateral breast cancer in Indonesia population. The competing risk factors were investigated using both univariate and multivariate regression analyses. In addition, the relation with progression-free survivals and time to distant metastasis were analyzed. Our study has limitations including the relatively shorter median follow-up as well as limitations associated with the retrospective hospital-based study design. The use of different criteria and methods in detecting contralateral breast cancer along with the duration of follow-up might contribute to the different range of the incidence. In addition, bilateral breast cancer has been associated with accumulation of genetic alterations due to biallelic sporadic events or genetic predisposition due to germline mutations that are not specifically analyzed in this study.

## Ethics approval

The study has been conducted following ethical principles according to the Declaration of Helsinki 1964 and the protocol has been approved by the Medical and Health Research Ethics Committee of the Faculty of Medicine, Public Health and Nursing - Universitas Gadjah Mada Yogyakarta (1143/EC/2017 and 1049/EC/2018).

## Sources of funding

This study was supported by grants from 10.13039/501100012521Universitas Gadjah Mada to SLA (RTA Nr 2488/2020 and DaMas Nr 133/2020) and NUS-UGM-Tahir Foundation to SLA (1/2020).

## Author contribution

SLA designed the study. SLA, DP, WSA collected and analyzed the data. SLA wrote the first draft with critical feedback from WAH and TA. All authors agreed on the final version of the manuscript draft.

## Trial registry number

1. Name of the registry: ISRCTN registry.

2. Unique Identifying number or registration ID: ISRCTN13788093.

3. Hyperlink to the registration (must be publicly accessible): http://www.isrctn.com/ISRCTN13788093.

## Guarantor

SLA, Universitas Gadjah Mada.

## Consent to publish

Consent for data presentation and publication has been obtained from all authors.

## Provenance and peer review

Not commissioned, externally peer reviewed.

## Declaration of competing interest

All authors have declared that no potential conflict of interest exists.

## Abbreviations

JCC: American Joint Committee on Cancer
BBC: bilateral breast cancer
CI: Confidence interval
EC: Ethical Clearance
ER: Estrogen Receptor
Her2: human epidermal growth factor receptor-2
mSBR: modified Bloom and Richardson system
MBC: metachronous bilateral breast cancer
MRI: Magnetic Resonance Imaging
N: Node status
OR: Odds Ratio
PR: Progesterone Receptor
SBC: synchronous bilateral breast cancer
SD: Standard deviation
T: Tumor size
UBC: unilateral breast cancer
